# Heterogeneity and the determinants of multidimensional health transition among Chinese middle-aged and older people: a person-centered approach

**DOI:** 10.3389/fmed.2025.1563419

**Published:** 2025-07-25

**Authors:** Linglong Ye, Zhengman Wu, Yi-Wei Kao, Jianping Zhu, Mingchih Chen, Ben-Chang Shia, Lei Qin

**Affiliations:** ^1^School of Public Affairs, Xiamen University, Xiamen, China; ^2^Department of Applied Statistics and Information Science, Ming Chuan University, Taoyuan, Taiwan; ^3^National Institute for Data Science in Health and Medicine, Xiamen University, Xiamen, China; ^4^Data Mining Research Center, Xiamen University, Xiamen, China; ^5^School of Management, Xiamen University, Xiamen, China; ^6^Graduate Institute of Business Administration, College of Management, Fu Jen Catholic University, New Taipei City, Taiwan; ^7^Artificial Intelligence Development Center, Fu Jen Catholic University, New Taipei City, Taiwan; ^8^School of Statistics, University of International Business and Economics, Beijing, China; ^9^Dong Fureng Institute of Economic and Social Development, Wuhan University, Wuhan, China

**Keywords:** aging, health transition, heterogeneity, multidimensional health, repeated-measures latent class analysis

## Abstract

**Introduction:**

Previous health transition patterns studies only focused on biological and psychological dimensions, and overlooked social dimension. By combining biological, psychological, and social health dimensions, this study aimed to examine heterogeneous transition patterns and assessed their determinants among Chinese middle-aged and older adults.

**Methods:**

Four waves of longitudinal data in 2011–2012, 2013, 2015, and 2018 from the China Health and Retirement Longitudinal Study was adopted. A total of 6,161 adults aged ≥45 years with no missing data on three dimensions were included. The repeated-measures latent class analysis as a person-centered approach was used to estimate transition patterns, followed by multinomial logistic regression to assess determinants.

**Results:**

This study highlighted five health transition groups, including “social participation slightly improving followed by deterioration” (SP-ID, 23.62%), “cognitive status deteriorating gradually and lacking social participation” (CS-DG&L-SP, 17.97%), “ADLs deteriorating followed by a slight improvement and lacking social participation” (ADLs-DI&L-SP, 9.14%), “died in the follow-up period” (DIFP, 11.65%), and “sustainedly healthy” (SH, 37.62%). Using the SH group as the reference group, sex, education, job, type of residence, and region have different impact on different patterns. Men were more likely to be in the SP-ID and DIFP groups, while women were more likely to be in the CS-DG&L-SP and ADLs-DI&L-SP groups. Being not in marriage was related to the SP-ID, CS-DG&L-SP, and DIFP groups. Those living in the central region and those having smoking and drinking habits less tended to fall into the SP-ID group. The retired, non-smokers, and drinkers even less tended to be in the CS-DG&L-SP group. Those living in the eastern region, those drinking alcohol appropriately, and those with higher income less tended to be in the ADLs-DI&L-SP group. Being uninsured by public insurance and having lower income were associated with the DIFP group.

**Conclusion:**

Integrating the social dimension with physical and psychological dimensions enhanced our understanding of the heterogeneous health transition patterns of middle-aged and older people. These findings provide valuable evidence for promoting healthy aging targets for different groups of the aging population.

## 1 Introduction

As global fertility and mortality decline simultaneously, the global population ages, with the proportion of people aged ≥65 expected to rise from 10% in 2022 to 16% in 2050 (1). This phenomenon is also occurring in developing countries, particularly in China, which is transitioning to an aged society at an incredible rate, and leapfrogging to have the world’s largest number of older people, with the number of people ≥65 expected to increase from 14% in 2015 to 26% in 2050 (2). However, longer life does not mean better health (3). In China, older people’s health is also becoming a serious challenge, and the aging population has unprecedented health needs, leading to huge healthcare expenditures. Therefore, it is imperative to comprehensively and accurately assess the health status and development trends of the middle-aged and elderly in China.

The World Health Organization defines health as “a state of physical, mental, and social well-being, not just the absence of disease and infirmity” (4). The widely accepted modern medical model also emphasizes the combined effect of the biological, psychological, and social dimensions (5). There is increasingly concerned with an integrated perspective across multiple dimensions, particularly the heterogeneous state of individual health resulting from the interaction of each dimension. However, the heterogeneity of multidimensional health transition patterns among middle-aged and older adults has not been studied properly, and previous studies still focus mainly on physical and psychological health (6, 7).

“Healthy aging” is relevant to everyone (8). To understand the multidimensional nature of individual health, a person-centered approach should be used, which differs from the traditional variable-centered approach, to distinguish heterogeneity in groups by considering the individual’s characteristics (9). Researchers use person-centered methods such as latent class analysis (LCA) and latent transition analysis to explain population heterogeneity by analyzing cross-sectional and short-term longitudinal data (10, 11). In addition, mixed models such as growth mixture modeling, which are based on longitudinal data spanning longer times, provide a dynamic perspective on older people’s health (7, 9). Nevertheless, most studies have focused on single-dimensional health trajectories (12, 13), with fewer studies having separately observed health transfer patterns of older adults from a more multidimensional perspective (14–16). Developing the repeated-measures LCA (RMLCA) on the LCA model allows for the simultaneous observation of changes in multidimensional indicators over time, allowing for a more intuitive study of health transfer patterns in older people (15, 17, 18).

Moreover, the determinants of ternary-dimensional health transition patterns should be examined to promote the vision of healthy aging achievement. Andersen’s behavioral model of health services use is a widely recognized model of health influences in which predisposing, enabling, and need factors influence individual health outcomes and health behaviors are considered (19, 20).

This study aimed to integrate physical, psychological, and social health indicators to identify potential heterogeneous multidimensional health transition patterns among middle-aged and older Chinese people. In addition, this study assessed the impact of predisposing factors, enabling factors, and health behaviors on different health transition patterns.

## 2 Methods

### 2.1 Study designs and samples

This study adopted longitudinal data from the China Health and Retirement Longitudinal Study (CHARLS). The CHARLS is a nationally representative longitudinal survey of residents in mainland China aged ≥45 and has been conducted every 2 to 3 years since 2011 (21). It provides a wide range of information on health conditions, socioeconomic status, and deaths occurring during the survey period. Each CHARLS wave has received ethical approval from the Institutional Review Board at Peking University. It uses a probability-proportional-to-size sampling with a stratified (by per-capita gross domestic product of rural counties and urban districts) multistage (county/district-village/community-household) design (22). Moreover, the Harmonized CHARLS data is provided to ensure the adoption of best practices and international comparisons. More detailed information on the study design and high quality of CHARLS data have been reported elsewhere (6, 23, 24).

**Figure 1:**
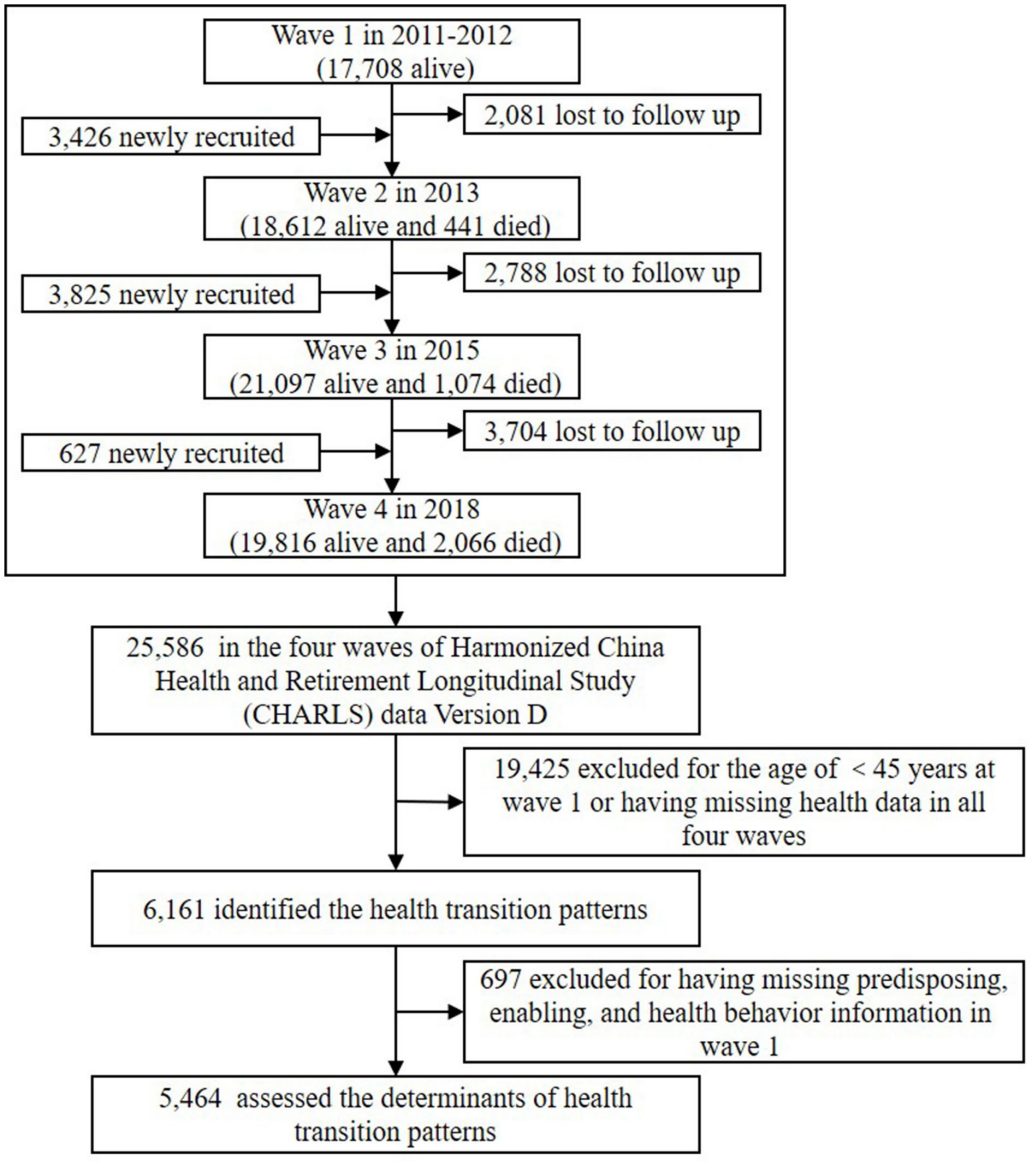
Flow chart of the Harmonized CHARLS data Version D follow-up and the sample selection of this study.

The longitudinal dataset in this study comprised all four waves of Harmonized CHARLS data Version D published in June 2021, with waves 1 to 4 in 2011–2012, 2013, 2015, and 2018, respectively (25). Given the potentially substantial impact of the COVID-19 pandemic and relevant government policies on individual multidimensional health in the 2020 survey, this study did not include the wave of 2020. The flow chart of the Harmonized CHARLS data Version D follow-up and the sample selection of this study is presented in Figure 1. Those aged ≥45 years old at wave 1 were included. To reduce the impact of survival bias, we included those who died during the study period (15). Respondents with missing health data were excluded, yielding 6,161 respondents aged ≥45 years to identify heterogeneous groups of health transition patterns, from which 5,464 respondents with complete information on predisposing, enabling, and health behavior information were included in the analyses of impact factors on health transition patterns.

### 2.2 Assessment of multidimensional health

This study used three physical, psychological, and social health indicators at each of the four waves to measure heterogeneous transition patterns. Given the RMLCA model applied in this study, we treated death as a category of health indicators rather than as another indicator, death, which would be over certainty and therefore not be able to result in a solution of the model (15).

Physical health status was measured by six activities of daily living (ADLs), including dressing, bathing and showering, eating, getting in and out of bed, using the toilet, and controlling urination and defecation (26). The assessment of ADLs was categorized into three categories: (1) no difficulties, corresponding to those without any assistance in any one of ADLs; (2) with difficulties, corresponding to those requiring any assistance in any activity; (3) being dead.

Psychological health status was examined by the Telephone Interview of Cognitive Status (TICS) (27). Although cognitive symptoms are often viewed as an indication of non-psychiatric disorders referring to functional health, the National Institute of Mental Health, the lead federal agency for research on mental disorders, called for recognition that cognition is in fact a symptom of psychiatric disorders (28, 29). Hence, we regarded cognitive status as psychological health status. In harmonized CHARLS, the TICS includes 10 items with scores ranging from 0 to 10: a test of serial subtractions of 7 from 100 up to five times, today’s date (year, month, day) and day of the week, and ability to draw a sign picture. A higher TICS score indicates better cognitive function. Those with a score less than a median of 8 were deemed as having cognitive problems. Therefore, the measure of TICS was defined as no cognitive problems, having cognitive problems, and being dead.

Social health status was assessed by social participation (SP) in social activities (30). The measure of SP was based on the six sub-questions of the same question about social activities. These questions indicated whether the respondent participated in any social activities in the past month: (1) interacted with friends; (2) played Ma-jong, chess, or cards, or went to a community club; (3) participated in sports, social, or other club activity; (4) participated in a community-related organization; (5) did voluntary or charity work; (6) attended an educational or training course. If the respondent did not participate in any of above social activities, they were deemed as lacking social engagement (31). Hence, the measure of SP was defined as not lacking SP, lacking SP, and being dead.

### 2.3 Covariates

In this study, all the covariates were measured in the first wave and were divided into three determinants based on Andersen’s behavioral model: predisposing, enabling, and health behaviors (19, 20). The predisposing determinants consisted of age (45–54, 55–64, or ≥65 years), sex (male or female), education (illiterate, literate or primary school, or junior high and above), job (agriculture, non-agriculture, retired, or others), marital status (in marriage or not in marriage). The measurement of the job about “others” included unemployed and have never worked. The enabling determinants comprised living arrangement (living alone or living with others), type of residence (urban or rural), region (eastern, central, western, or northeast), public health insurance (yes or no), household income per capita (<1,000 yuan, or ≥1,000 yuan). The divisions of regions were based on the 2011 version of China’s economic regional divisions (32). The health behaviors included smoking behavior (yes or no) and drinking behavior (yes or no). Detailed information about the measurements of covariates is presented in Table 1.

**Table 1:** The values assigned to indicators.

Health dimension	Variable	Value assignment
Physical health status	The activities of daily living (ADLs)	1 = No difficulty; 2 = With difficulties; 3 = Being dead.
Psychological health status	The Telephone Interview of Cognitive Status battery (TICS)	1 = No cognitive problems; 2 = Having cognitive problems; 3 = Being dead
Social health status	The social participation	1 = No lacking social participation; 2 = Lacking social participation; 3 = Being dead.

Covariate	Variable	Value assignment
Predisposing	Age	45–54, 55–64, or 65 + years
	Sex	Male or Female
	Education	Illiterate, Literate or primary school, or Junior high and above
	Job	Agriculture, Non-agriculture, Retired, or Others
	Marital status	In marriage or Not in marriage
Enabling	Living arrangement	Living alone, or Living with others
	Type of residence	Urban or Rural
	Region	Eastern, Central, Western, or Northeast
	Public health insurance	Yes or No
	Household income per capita	Lower than 1,000 yuan, or Higher than 1,000 yuan
Health behaviors	Smoke behavior	Yes or No
	Drink behavior	Yes or No

### 2.4 Statistical analyses

The statistical analyses in this study involved two steps. The first step identified the health transition patterns by RMLCA based on the three indicators at each of the four waves. The RMLCA model applied LCA to classify the changes during several times into different subgroups and enabled defining of the particular patterns of discontinuous development (33), which corresponded to a certain transition pattern of multidimensional health in this study. When a small number of indicators are measured three or more times, previous studies have pointed out that the RMLCA approach works best (17). As the basis of RMLCA, LCA is a person-centered method suitable for addressing heterogeneity (6). In an LCA model, those with similar performance in observable indicator features are categorized into the same unobservable subgroup, which is related to a latent class (6, 15, 17). Estimated indicator–response probabilities and class membership probabilities were reported. By comparing indicator–response probabilities among different classes, the labels of latent health transition patterns were based on the specific characteristics of certain indicator–response probabilities which different from other classes (17). The models with 2–10 classes were conducted to find the best-fitting model. The performance of the models was estimated by Bayesian information criterion (BIC), adjusted BIC (aBIC), and consistent Akaike’s information criterion (cAIC), where a lower value indicates a better performance. In addition, entropy with a range of 0 to 1 was used to measure the classification quality of the models, and a higher entropy value generally indicates a better separate of the latent classes.

The second step was to conduct a multinomial logistic regression analysis to examine how predisposing, enabling, and health behaviors impact different health transition patterns. All the statistical analyses were performed using R, version 4.2.2. A *p*-value<0.05 was regarded as statistically significant.

### 2.5 Sensitivity analyses

Three sensitivity analyses were performed to verify the robustness of the results. First, we replaced ADLs with instrumental activities of daily living (IADLs) as a measure of physical health status and reconducted RMLCA and multinomial logistic regression analyses. In harmonized CHARLS data, IADLs include the following items: managing money, taking medications, shopping for groceries, preparing meals, cleaning the house, and making phone calls. Previous studies have pointed out ADL and IADL were both widely used in the measurement of functional disability, related to physical health status in this study (34). However, previous studies have suggested that IADL disability is the prior stage of ADL disability (35). Therefore, we used ADLs to measure relatively stable physical health status.

Second, we included those respondents with complete health information or only having one missing health information in the analyze of latent health transition patterns. Given it is common to use the complete case analysis method including only samples with complete data for analysis in previous studies of the CHARLS database (36, 37), we excluded those respondents with missing health data in the identification of latent health transition patterns.

Three, using multivariable logistic regression, we examined the effect of predisposing, enabling, and health behaviors on all the classes of the best-fitting model, which may reflect a more comprehensive picture of the relationship between covariates and health status trajectories. However, several classes of the best-fitting model had a small sample size, which may result in biased estimation. The parameters of logistic regression are often estimated using maximum likelihood estimation, which is prone to bias when the sample size is small (38). General guidelines for the minimum number of events per variable (EPV) are required in logistic regression. It has been demonstrated that when the EPV values are ≥10, it does not seriously impact the results (39). Hence, to ensure that the results were statistically significant, we combined those classes with a small number of samples (17).

## 3 Results

### 3.1 Identification of latent health transition patterns

This study included 6,161 respondents aged ≥45 years with complete health information in the identification of latent health transition patterns, and their multidimensional health status of respondents during the four waves from 2011 to 2018 is shown in Table 2. The proportions of the responses with ADL difficulties were 12.60, 13.18, 15.47, and 14.28% at four waves, and the trend went up at wave 2 and 3 but went down at wave 4. The proportions of having cognitive problems during the four waves (31.67, 29.62, 28.97, and 37.12%, respectively) firstly fell at wave 2 and 3, and rose obviously at wave 4. The trend of lacking social participation (50.22, 40.04, 43.22, and 45.28%, respectively) declined at wave 2 and then increased at wave 3 and 4. The proportions of death were 3.90, 7.13, and 11.65% at wave 2–4, respectively.

**Table 2:** Multidimensional health status of respondents during the four waves from 2011 to 2018.

Health dimension	2011–2012^a^	2013^a^	2015^a^	2018^a^
Physical health status				
ADL with no difficulty	5,385 (87.40%)	5,109 (82.92%)	4,769 (77.41%)	4,563 (74.06%)
ADL with difficulties	776 (12.60%)	812 (13.18%)	953 (15.47%)	880 (14.28%)
Being dead	–	240 (3.90%)	439 (7.13%)	718 (11.65%)
Psychological health status				
No cognitive problems	4,210 (68.33%)	4,096 (66.48%)	3,937 (63.90%)	3,156 (51.23%)
Having cognitive problems	1,951 (31.67%)	1,825 (29.62%)	1,785 (28.97%)	2,287 (37.12%)
Being dead	–	240 (3.90%)	439 (7.13%)	718 (11.65%)
Social health status				
No lacking social participation	3,067 (49.78%)	3,454 (56.06%)	3,059 (49.65%)	2,653 (43.06%)
Lacking social participation	3,094 (50.22%)	2,467 (40.04%)	2,663 (43.22%)	2,790 (45.28%)
Being dead	–	240 (3.90%)	439 (7.13%)	718 (11.65%)

^a^*n* (%).

**Figure 2:**
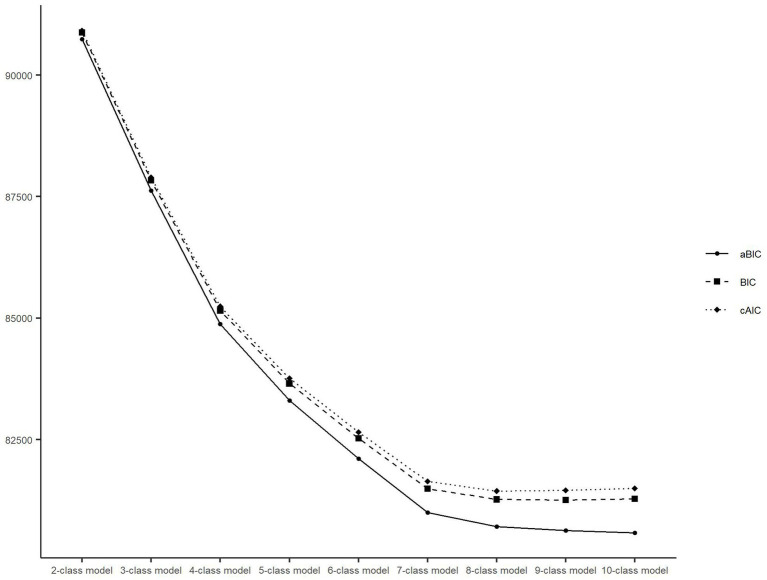
Latent class model and fit indices. BIC, Bayesian information criterion; aBIC, adjusted Bayesian information criterion; cAIC, consistent Akaike’s information criterion.

**Figure 3:**
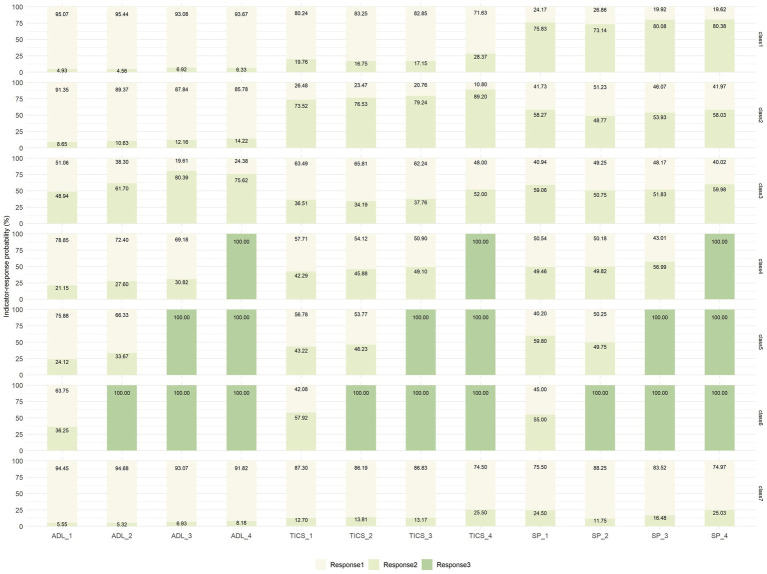
Latent class probability and indicator–response probabilities for the seven classes of health transition patterns. ADL, activities of daily living; TICS, Telephone Interview of Cognitive Status; SP, social participation. For ADL: response 1 = no difficulty, response 2 = with difficulties, response 3 = being dead; for TICS: response 1 = no cognitive problems, response 2 = having cognitive problems, response 3 = being dead; for SP: response 1 = no lacking SP, response 2 = lacking SP, response 3 = being dead. ADL_1–4: measured during the Wave 1–4. TICS and SP are the same as ADL.

The performance of the different class models is shown in Figure 2. The BIC, aBIC, and cAIC values declined as more latent classes were added. However, after the seven-class model, the downward trend gradually leveled off. The entropy values of the models with 2–10 classes were all over 0.7, corresponding to 1.000, 0.766, 0.783, 0.770, 0.782, 0.789, 0.760, 0.746, and 0.745, respectively. In addition, the classifications identified by the seven-class model could more adequately represent heterogeneous transition patterns of multidimensional health, including the maintenance of a relatively healthy state, changes in different health indicators, and death at different periods (Figure 3). Therefore, we chose the seven-class model as the best model.

### 3.2 Characteristics of health transition patterns

The labels of latent health transition patterns were defined by comparing indicator–response probabilities (Figure 3). The first class (*n* = 1,455, 23.62%) was characterized by a high probability of lacking SP, showing a slight decrease followed by an increase (75.83, 73.14, 80.08, and 80.38% in waves 1 to 4, respectively). Meanwhile, the probabilities of having ADLs difficulties (4.93, 4.56, 6.92, and 6.33%, in waves 1 to 4, respectively) and cognitive problems (19.76, 16.75, 17.15, and 28.37% in waves 1 to 4, respectively) were consistently low. Hence, this group was labeled “SP slightly improving followed by deterioration” (SP-ID).

The second class (*n* = 1,107, 17.97%) tended to have high probabilities of having cognitive problems (73.52, 76.53, 79.24, and 89.20% in waves 1 to 4, respectively), which tended to increase during the study period. The probability of ADLs difficulties (8.65, 10.63, 12.16, and 14.22% in waves 1 to 4, respectively) remained relatively low, but the probabilities of lacking SP also fluctuated around 50% (58.27, 48.77, 53.93, and 58.03% in waves 1 to 4, respectively). Thus, this group was labeled “cognitive status deteriorating gradually and lacking SP” (CS-DG&L-SP).

The third class (*n* = 563, 9.14%) was prone to high probabilities of having ADLs impairment and a trend of a consistent increase followed by a decrease over the four periods, peaking in wave 3 (48.94, 61.70, 80.39, and 75.62% in waves 1 to 4, respectively). At the same time, the probabilities of having cognitive problems increased significantly (36.51, 34.19, 37.76, and 52.00% in waves 1 to 4, respectively) and lacking SP consistently exceeded 50% (59.06, 50.75, 51.83, and 59.98%, in waves 1 to 4, respectively). Therefore, this group was named “ADLs deteriorating followed by a slight improvement and lacking SP” (ADLs-DI&L-SP).

The fourth, fifth, and sixth classes with a small sample size (*n* = 279, 4.53%, *n* = 199, 3.23%, and *n* = 240, 3.90%, respectively) corresponded to those who died during the follow-up in three waves and represented the outcomes of succumbing to death. Considering the guidelines of the EPV values, we combined the three classes into one group labeled “died in the follow-up period” (DIFP), which accounted for 11.65% (n = 682) of the whole study population. Therefore, this study adopted five groups of health transition patterns after merging the death samples from the seven-class model.

For those respondents in the last class (*n* = 2,318, 37.62%), the likelihoods of having ADLs difficulties (5.55, 5.32, 6.93, and 8.18% in waves 1 to 4, respectively), having cognitive problems (12.70, 13.81, 13.17, and 25.50% in waves 1 to 4, respectively), lacking SP (24.50, 11.75, 16.48, and 25.03% in waves 1 to 4, respectively) were comparatively low and stable throughout the four waves. Therefore, this group was labeled “sustainedly healthy” (SH).

**Figure 4:**
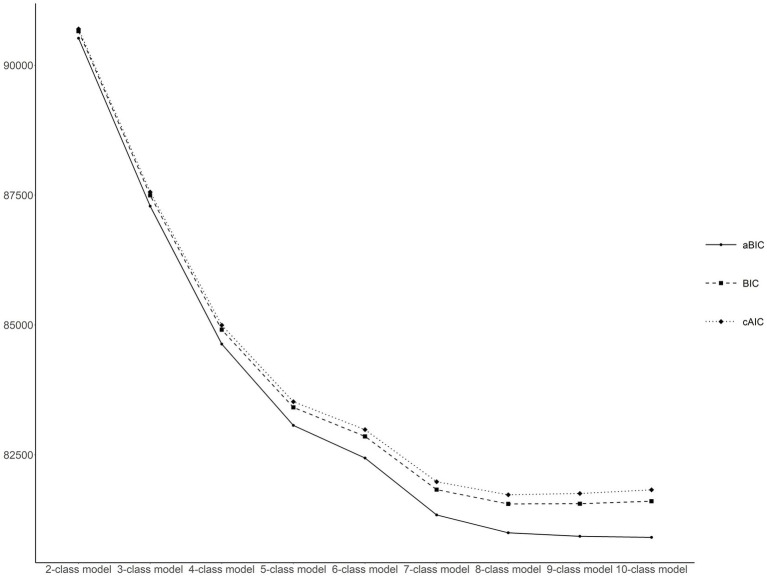
Latent class model and fit indices after replacing health variables. BIC, Bayesian information criterion; aBIC, adjusted Bayesian information criterion; cAIC, consistent Akaike’s information criterion.

**Figure 5:**
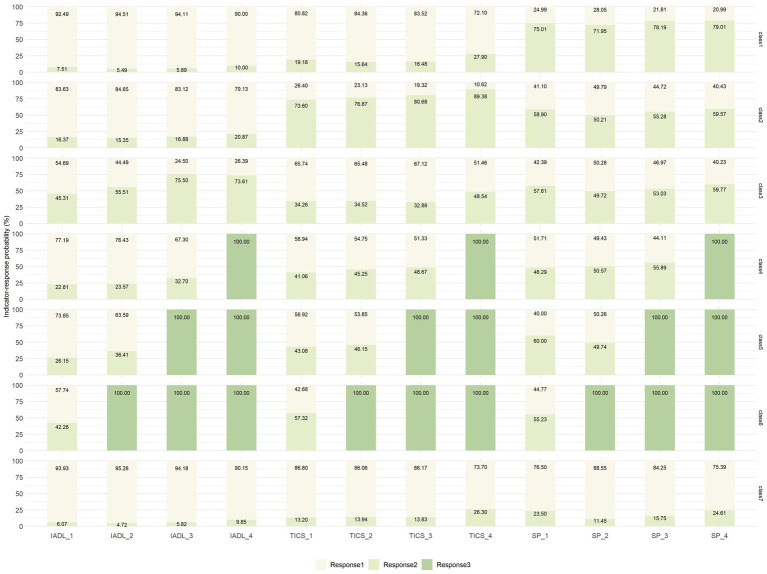
Latent class probability and indicator-response probabilities after replacing health variables. ADL, activities of daily living; TICS, Telephone Interview of Cognitive Status; SP, social participation; For IADL: response 1 = no difficulty, response 2 = with difficulties, response 3 = being dead; for TICS: response 1 = no cognitive problems, response 2 = having cognitive problems, response 3 = being dead; for SP: response 1 = no lacking SP, response 2 = lacking SP, response 3 = being dead. IADL_1–4: measured during the Wave 1–4. TICS and SP are the same as IADL.

After replacing physical health indicators, the performance of the RMLCA models with 2–10 classes was reestimated, which showed a similar downward trend as before (Figure 4). The entropy values of the reestimated models with 2–10 classes were also over 0.7, related to 1.000, 0.772, 0.789, 0.768, 0.765, 0.773, 0.749, 0.748, and 0.732, respectively. Given the similar results to the original sample, the seven-class model was chosen again. In addition, each health transition pattern was almost identical in characteristics and proportion to the original RMLCA classification (Figure 5). Hence, according to the different characteristics of indicator–response probabilities among different classes, the labels of reestimated seven classes could be defined to the similar labels of raw seven classes. Moreover, the proportions of reestimated seven classes (27.76, 17.49, 7.46, 4.28, 3.18, 3.89, 35.93%) were close to that of raw seven classes.

**Figure 6:**
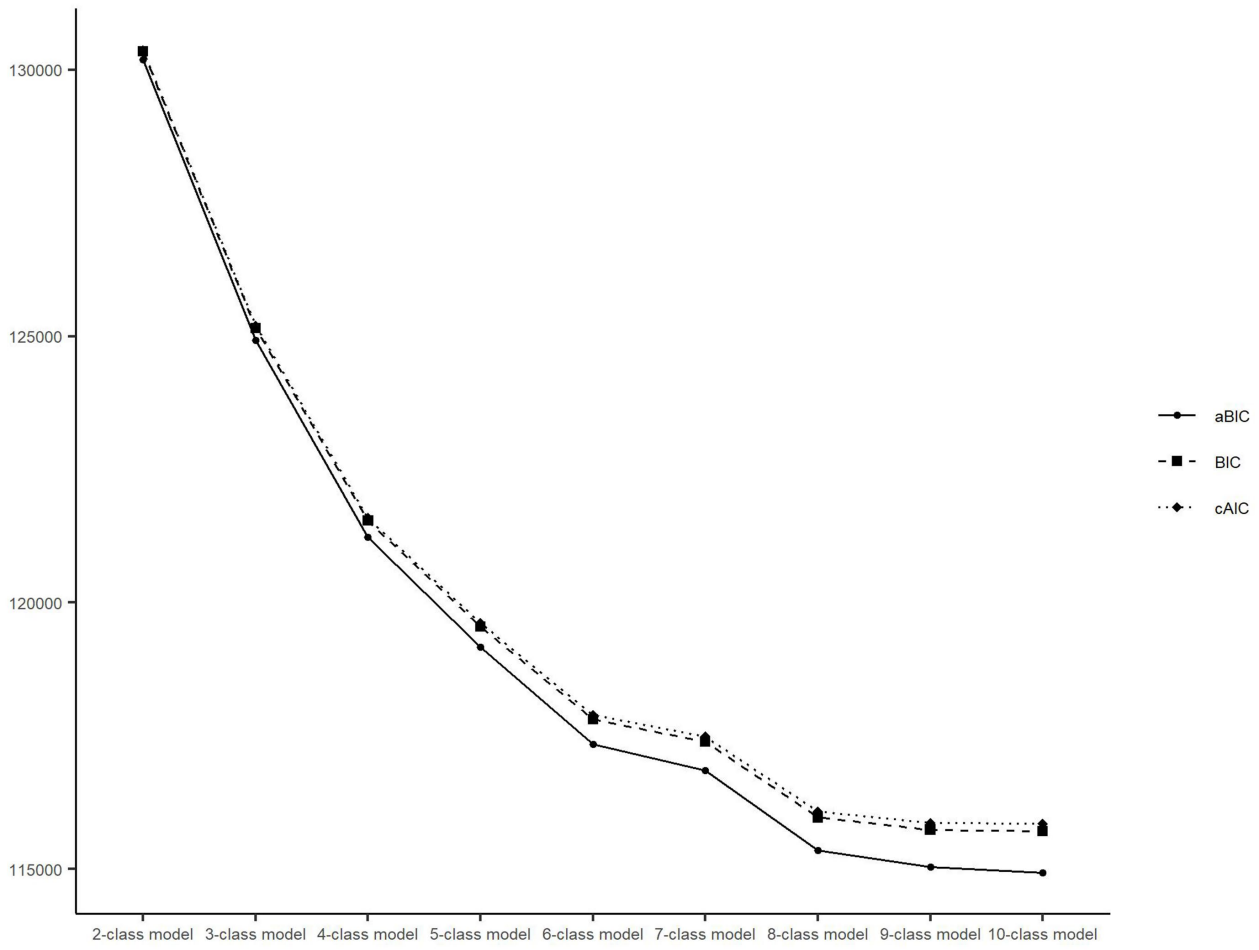
Latent class model and fit indices after including respondents with complete health information or only having one missing health information. BIC, Bayesian information criterion; aBIC, adjusted Bayesian information criterion; cAIC, consistent Akaike’s information criterion.

**Figure 7:**
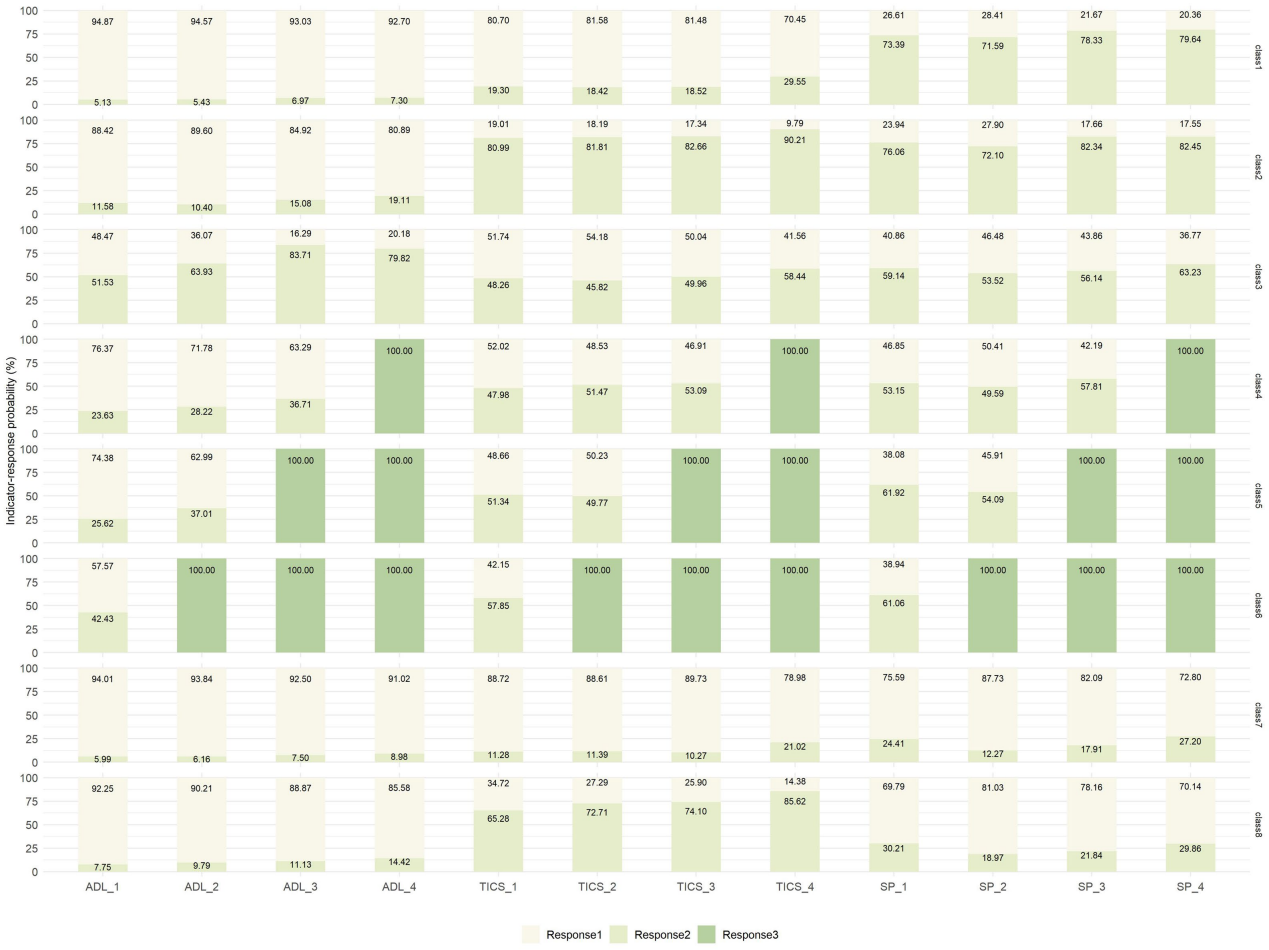
Latent class probability and indicator–response probabilities after respondents with including complete health information or only having one missing health information. ADL, activities of daily living; TICS, Telephone Interview of Cognitive Status; SP, social participation; For IADL: response 1 = no difficulty, response 2 = with difficulties, response 3 = being dead; for TICS: response 1 = no cognitive problems, response 2 = having cognitive problems, response 3 = being dead; for SP: response 1 = no lacking SP, response 2 = lacking SP, response 3 = being dead. IADL_1–4: measured during the Wave 1–4. TICS and SP are the same as IADL.

After including those respondents with complete health information or only having one missing health information, we covered 8,780 respondents, which was more than the original sample size of 6,161. The performance of the RMLCA models with 2–10 classes was reestimated again (Figure 6). It showed the downward trend gradually leveled off after the eight-classes model rather than seven-class model as before. The entropy values of those models were also over 0.7, corresponding to 1.000, 0.790, 0.805, 0.773, 0.782, 0.751, 0.757, 0.746, and 0.738, respectively. The indicator–response probabilities of eight class were shown in Figure 7. Except for the last class (*n* = 1,147, 13.06%), the characteristics of indicator–response probabilities among the other seven classes were similar to the raw seven classes. In addition, the proportions of reestimated first, fourth, fifth, and sixth classes (23.05, 4.16, 3.20, and 3.86%) were close to raw related classes, and the proportions of reestimated second and seventh classes (14.10 and 28.21%) were lower than raw related classes, while the proportion of reestimated third class (10.35%) was higher than raw related classes. Although the larger sample size led to a greater number of classes of health transition patterns, the structures and partitions of health transition patterns were still similar to the original results.

### 3.3 Associations between latent health transition patterns and covariates

Table 3 presents the characteristics of the respondents in different health transition groups in 2011. There were significant differences in predisposing, enabling, and health behaviors. For predisposing factors, the proportion of 45–54 years old (40.1%) was the highest among all age groups, and the proportion of elderly people aged 65 and above was higher in the other groups than in the SH group. Males and females accounted for 46.58 and 53.42% respectively, but the proportions of males in CS-DG&L-SP and ADLs-DI&L-SP groups (38.4 and 44.8%, respectively) were lower than that of females (61.6 and 55.2%, respectively). Nearly a tenth of the respondents (9.8%) were illiterate, which proportions in CS-DG&L-SP and DIFP groups (22.6 and 22.4%, respectively) were higher than other groups. The job of approximately two fifths of the respondents (39.5%) were agriculture, and its proportion in CS-DG&L-SP group (51.7%) was the highest among all health transition groups. Only 9% of the respondents were not in marriage, which proportion in DIFP group (21.6%) was higher than other groups. For enabling factors, 4.5% live alone, and its proportion in DIFP group (11.0%) was the only one higher than the mean rate of all samples. Almost three-fifths (58.3%) were rural, and its proportion in SH groups (49.4%) were the only one lower than the sample mean. The proportion of all samples living in the eastern (31.9%) was highest among all regions, but region with the highest proportion in the CS-DG&L-SP, ADLs-DI&L-SP, and DIFP groups was western (36.0, 35.2, and 35.1%, respectively). Only 6.2% did not have public health insurance, which proportion in DIFP group (9.2%) was the higher than other groups. For health behaviors, about one-third of the respondents smoked, and its proportion in DIFP group (40.5%) was the highest among all groups. Over one-third of the respondents drank (34.1%), and its proportion in SH and SP-ID group (39.2 and 34.7%, respectively) was higher than the mean rate of all samples.

**Table 3:** Baseline characteristics of respondents in the five latent health transition groups.

Characteristic	Overall, *N* = 5,464^a^	SH, *N* = 2,071^a^	SP-ID, *N* = 1,291^a^	CS-DG&L-SP, *N* = 972^a^	ADLs-DI&L-SP, *N* = 500^a^	DIFP, *N* = 630^a^	*p*-value^b^
Predisposing factor							
Age							<0.001
45–54	2,192 (40.1%)	1,006 (48.6%)	552 (42.8%)	418 (43.0%)	129 (25.8%)	87 (13.8%)	
55–64	2,181 (39.9%)	771 (37.2%)	553 (42.8%)	405 (41.7%)	248 (49.6%)	204 (32.4%)	
65+	1,091 (20.0%)	294 (14.2%)	186 (14.4%)	149 (15.3%)	123 (24.6%)	339 (53.8%)	
Sex							<0.001
Male	3,026 (55.4%)	1,210 (58.4%)	781 (60.5%)	373 (38.4%)	224 (44.8%)	438 (69.5%)	
Female	2,438 (44.6%)	861 (41.6%)	510 (39.5%)	599 (61.6%)	276 (55.2%)	192 (30.5%)	
Education							<0.001
Illiterate	536 (9.8%)	54 (2.6%)	75 (5.8%)	220 (22.6%)	46 (9.2%)	141 (22.4%)	
Literate or primary school	2,459 (45.0%)	729 (35.2%)	594 (46.0%)	526 (54.1%)	291 (58.2%)	319 (50.6%)	
Junior high and above	2,469 (45.2%)	1,288 (62.2%)	622 (48.2%)	226 (23.3%)	163 (32.6%)	170 (27.0%)	
Job							<0.001
Agriculture	2,159 (39.5%)	645 (31.1%)	583 (45.2%)	503 (51.7%)	220 (44.0%)	208 (33.0%)	
Non-agriculture	1,495 (27.4%)	744 (35.9%)	401 (31.1%)	209 (21.5%)	64 (12.8%)	77 (12.2%)	
Retired	1,702 (31.1%)	649 (31.3%)	282 (21.8%)	246 (25.3%)	201 (40.2%)	324 (51.4%)	
Others	108 (2.0%)	33 (1.6%)	25 (1.9%)	14 (1.4%)	15 (3.0%)	21 (3.3%)	
Marital status							<0.001
In marriage	4,973 (91.0%)	1,945 (93.9%)	1,201 (93.0%)	877 (90.2%)	456 (91.2%)	494 (78.4%)	
Not in marriage	491 (9.0%)	126 (6.1%)	90 (7.0%)	95 (9.8%)	44 (8.8%)	136 (21.6%)	
Enabling factor							
Living arrangement							<0.001
Alone	246 (4.5%)	80 (3.9%)	41 (3.2%)	37 (3.8%)	19 (3.8%)	69 (11.0%)	
With others	5,218 (95.5%)	1,991 (96.1%)	1,250 (96.8%)	935 (96.2%)	481 (96.2%)	561 (89.0%)	
Type of residence							<0.001
Rural	3,188 (58.3%)	1,023 (49.4%)	785 (60.8%)	657 (67.6%)	343 (68.6%)	380 (60.3%)	
Urban	2,276 (41.7%)	1,048 (50.6%)	506 (39.2%)	315 (32.4%)	157 (31.4%)	250 (39.7%)	
Region							<0.001
Eastern	1,742 (31.9%)	707 (34.1%)	459 (35.6%)	278 (28.6%)	106 (21.2%)	192 (30.5%)	
Central	1,578 (28.9%)	650 (31.4%)	333 (25.8%)	260 (26.7%)	171 (34.2%)	164 (26.0%)	
Western	1,645 (30.1%)	543 (26.2%)	355 (27.5%)	350 (36.0%)	176 (35.2%)	221 (35.1%)	
Northeast	499 (9.1%)	171 (8.3%)	144 (11.2%)	84 (8.6%)	47 (9.4%)	53 (8.4%)	
Public health insurance							0.016
Yes	5,123 (93.8%)	1,948 (94.1%)	1,217 (94.3%)	910 (93.6%)	476 (95.2%)	572 (90.8%)	
No	341 (6.2%)	123 (5.9%)	74 (5.7%)	62 (6.4%)	24 (4.8%)	58 (9.2%)	
Household income per capita							<0.001
<1,000	3,074 (56.3%)	1,017 (49.1%)	692 (53.6%)	569 (58.5%)	344 (68.8%)	452 (71.7%)	
1,000+	2,390 (43.7%)	1,054 (50.9%)	599 (46.4%)	403 (41.5%)	156 (31.2%)	178 (28.3%)	
Health behavior							
Smoke behavior							<0.001
Yes	1,841 (33.7%)	734 (35.4%)	429 (33.2%)	284 (29.2%)	139 (27.8%)	255 (40.5%)	
No	3,623 (66.3%)	1,337 (64.6%)	862 (66.8%)	688 (70.8%)	361 (72.2%)	375 (59.5%)	
Drink behavior							<0.001
Yes	1,863 (34.1%)	812 (39.2%)	448 (34.7%)	262 (27.0%)	127 (25.4%)	214 (34.0%)	
No	3,601 (65.9%)	1,259 (60.8%)	843 (65.3%)	710 (73.0%)	373 (74.6%)	416 (66.0%)	

SH, sustainedly healthy; SP-ID, SP slightly improving followed by deterioration; CS-DG&L-SP, Cognitive status deteriorating gradually and lacking SP; ADLs-DI&L-SP, ADLs deteriorating followed by a slightly improvement and lacking SP; DIFP, died in the follow-up period.

^a^*n* (%).

^b^Pearson’s Chi-squared test.

Table 4 presents the multinomial logistic regression results concerning the relationships between baseline characteristics and latent health transition groups. The SH group was considered the reference group in the multinomial logistic regression because it represented a relatively good and stable multidimensional health transition pattern during the study period. Except for living arrangement, other covariates have significantly impact on transition patterns. Especially, sex, education, job, type of residence, and region played different roles in different transition patterns.

**Table 4:** Associations of baseline characteristics in the five latent health transition groups by multivariable logistic regression.

Characteristic	SP-ID	CS-DG&L-SP	ADLs-DI&L-SP	DIFP
	OR (95% CI)	OR (95% CI)	OR (95% CI)	OR (95% CI)
Predisposing factor				
Age				
45–54	—	—	—	—
55–64	1.12 (0.95, 1.33)	0.85 (0.70, 1.03)	1.86 (1.44, 2.40)^***^	1.71 (1.28, 2.27)^***^
65+	1.03 (0.81, 1.31)	0.85 (0.65, 1.12)	2.25 (1.64, 3.11)^***^	5.28 (3.88, 7.19)^***^
Sex				
Male	—	—	—	—
Female	0.70 (0.58, 0.85)^***^	1.84 (1.46, 2.31)^***^	1.47 (1.12, 1.93)^**^	0.40 (0.31, 0.53)^***^
Education				
Illiterate	—	—	—	—
Literate or primary school	0.57 (0.39, 0.82)^**^	0.21 (0.15, 0.29)^***^	0.58 (0.38, 0.89)^*^	0.15 (0.11, 0.22)^***^
Junior high and above	0.38 (0.26, 0.56)^***^	0.06 (0.04, 0.08)***	0.28 (0.18, 0.44)***	0.07 (0.05, 0.10)***
Job				
Agriculture	—	—	—	—
Non-agriculture	0.73 (0.61, 0.88)^***^	0.67 (0.53, 0.83)^***^	0.54 (0.39, 0.74)^***^	0.69 (0.51, 0.95)^*^
Retired	0.53 (0.44, 0.65)^***^	0.64 (0.51, 0.79)^***^	1.13 (0.88, 1.45)	1.50 (1.17, 1.92)^**^
Others	0.91 (0.53, 1.57)	0.55 (0.28, 1.08)	1.33 (0.69, 2.58)	2.83 (1.50, 5.33)^**^
Marital status				
In marriage	—	—	—	—
Not in marriage	1.40 (1.00, 1.97)^*^	1.57 (1.10, 2.25)^*^	1.19 (0.76, 1.87)	2.63 (1.82, 3.80)^***^
Enabling factor				
Living arrangement				
Alone	—	—	—	—
With others	1.53 (0.96, 2.42)	1.49 (0.90, 2.49)	1.50 (0.79, 2.82)	1.25 (0.77, 2.04)
Type of residence				
Rural	—	—	—	—
Urban	0.79 (0.68, 0.93)^**^	0.64 (0.53, 0.76)^***^	0.46 (0.36, 0.58)^***^	0.72 (0.58, 0.89)^**^
Region				
Eastern	—	—	—	—
Central	0.77 (0.65, 0.93)^**^	1.07 (0.86, 1.32)	1.78 (1.35, 2.34)^***^	0.98 (0.76, 1.27)
Western	0.95 (0.79, 1.15)	1.57 (1.27, 1.94)^***^	1.96 (1.49, 2.58)^***^	1.37 (1.06, 1.75)^*^
Northeast	1.48 (1.14, 1.92)^**^	1.42 (1.03, 1.96)^*^	1.87 (1.25, 2.78)^**^	1.33 (0.90, 1.95)
Public health insurance				
Yes	—	—	—	—
No	1.01 (0.74, 1.37)	1.25 (0.89, 1.77)	0.88 (0.55, 1.40)	1.65 (1.14, 2.39)^**^
Household income per capita				
<1,000	—	—	—	—
1,000+	0.95 (0.81, 1.11)	0.97 (0.81, 1.16)	0.75 (0.59, 0.94)^*^	0.77 (0.61, 0.96)^*^
Health behavior				
Smoke behavior				
Yes	—	—	—	—
No	1.25 (1.05, 1.50)^*^	0.79 (0.63, 0.99)^*^	1.07 (0.82, 1.39)	0.95 (0.75, 1.20)
Drink behavior				
Yes	—	—	—	—
No	1.38 (1.16, 1.63)^***^	1.24 (1.01, 1.53)^*^	1.34 (1.04, 1.74)^*^	1.22 (0.97, 1.53)

The sustainedly healthy group is the reference group (*n* = 2071, 37.9%).

SP-ID, SP slightly improving followed by deterioration; CS-DG&L-SP, Cognitive status deteriorating gradually and lacking SP; ADLs-DI&L-SP, ADLs deteriorating followed by a slightly improvement and lacking SP; DIFP, died in the follow-up period; OR: odds ratio; CI, Confidence Interval.

^*^p < 0.05; ^**^p < 0.01; ^***^p < 0.001.

Age at baseline was positively associated with being classified in the ADLs-DI&L-SP, and DIFP groups. Compared to people aged 45–54, those over 55 were more likely to be in the DIFP and ADLs-DI&L-SP groups. Being a female was a protective factor for the SP-ID and DIFP groups (odds ratio [OR] = 0.70, confidence interval [CI]: 0.58–0.85 and OR = 0.40, CI: 0.31–0.53, respectively), but a risk factor for the CS-DG&L-SP and ADLs-DI&L-SP groups (OR = 1.84, CI: 1.46–2.31 and OR = 1.47, CI: 1.21–1.93, respectively) compared with males. Participants with a higher level of education were less likely to fall into the four non-healthy groups. In particular, those with an educational level of junior high and above were the least likely to experience deterioration in cognitive status (OR = 0.06, CI: 0.04–0.08). People working in non-agricultural sectors were less prone to health deterioration or death. Being retired at baseline was negatively associated with falling into the SP-ID and CS-DG&L-SP groups (OR = 0.53, CI: 0.44–0.65 and OR = 0.64, CI: 0.51–0.79, respectively). However, retirement or engagement in other works was positively associated with death during the follow-up period (OR = 1.50, CI: 1.17–1.92 and OR = 2.83, CI: 1.50–5.33, respectively). Those being not in marriage were prone to be assigned to the SP-ID, CS-DG&L-SP, and DIFP groups (OR = 1.40, CI: 1.00–1.97, OR = 1.57, CI: 1.10–2.25, and OR = 2.63, CI: 1.82–3.80, respectively).

Participants in urban areas were less likely to be classified as having health deterioration or succumbing to death than those in rural areas. Those in central regions tended to be less likely to be in the SP-ID group (OR = 0.77, CI: 0.65–0.93) and more likely to be in the ADLs-DI&L-SP group (OR = 1.78, CI: 1.35–2.34) than eastern regions. The likelihood of being in the CS-GD&L-SP, ADLs-DI&L-SP, and DIFP groups was higher in the western regions than in the eastern regions (OR = 1.57, CI: 1.27–1.94, OR = 1.96, CI: 1.49–2.58, and OR = 1.37, CI: 1.06–1.75, respectively). Furthermore, those living in the northeast regions had a higher likelihood of falling into the SP-ID, CS-DG&L-SP, and ADLs-DI&L-SP groups than those in the eastern regions (OR = 1.48, CI: 1.14–1.92, OR = 1.42, CI: 1.03–1.96, and OR = 1.87, CI: 1.25–2.78, respectively). Having public health insurance was associated with lower a risk of death during the follow-up period (OR = 1.65, CI: 1.14–2.39). Those with a household income per capita of ≥1,000 yuan were less likely to be in the ADLs-DI&L-SP and DIFP groups than in the SH group (OR = 0.75, CI: 0.59–0.94, and OR = 0.77, CI: 0.61–0.96, respectively).

Those who did not smoke were more likely to be in the SP-ID group but had a lower risk of falling into the CS-DG&L-SP group (OR = 1.25, CI: 1.05–1.50, OR = 0.79, CI: 0.63–0.99, respectively). People without drinking habits were more likely to experience health deterioration than those with drinking habits.

In the robustness analyses, the multinomial logistic regression for retaining seven classes showed stable associations between baseline characteristics and health transition classes, as presented in the (Tables 5, 6). In addition, based on the RMLCA of replacement physical health indicators, we ran a multinomial logistic regression on the classification of combined death samples and all health transition classes, respectively. Similar results were also observed (Tables 7–10). However, the relationship between marital status and the cognitive and social deterioration groups was no longer significant. In addition, the participants’ gender and residence in central regions did not significantly affect the IADLs-DI&L-SP group.

**Table 5:** Baseline characteristics of respondents in the seven latent health transition classes.

Characteristic	Overall, *N* = 5,464^a^	SH, *N* = 2,071^a^	SP-ID, *N* = 1,291^a^	CS-DG&L-SP, *N* = 972^a^	ADLs-DI&L-SP, *N* = 500^a^	DIED-2, *N* = 214^a^	DIED-3, *N* = 177^a^	DIED-4, *N* = 239^a^	*p*-value^b^
Predisposing factor									
Age									<0.001
45–54	2,192 (40.1%)	1,006 (48.6%)	552 (42.8%)	418 (43.0%)	129 (25.8%)	22 (10.3%)	32 (18.1%)	33 (13.8%)	
55–64	2,181 (39.9%)	771 (37.2%)	553 (42.8%)	405 (41.7%)	248 (49.6%)	59 (27.6%)	52 (29.4%)	93 (38.9%)	
65+	1,091 (20.0%)	294 (14.2%)	186 (14.4%)	149 (15.3%)	123 (24.6%)	133 (62.1%)	93 (52.5%)	113 (47.3%)	
Sex									<0.001
Male	3,026 (55.4%)	1,210 (58.4%)	781 (60.5%)	373 (38.4%)	224 (44.8%)	133 (62.1%)	118 (66.7%)	187 (78.2%)	
Female	2,438 (44.6%)	861 (41.6%)	510 (39.5%)	599 (61.6%)	276 (55.2%)	81 (37.9%)	59 (33.3%)	52 (21.8%)	
Education									<0.001
Illiterate	536 (9.8%)	54 (2.6%)	75 (5.8%)	220 (22.6%)	46 (9.2%)	64 (29.9%)	35 (19.8%)	42 (17.6%)	
Literate or primary school	2,459 (45.0%)	729 (35.2%)	594 (46.0%)	526 (54.1%)	291 (58.2%)	102 (47.7%)	85 (48.0%)	132 (55.2%)	
Junior high and above	2,469 (45.2%)	1,288 (62.2%)	622 (48.2%)	226 (23.3%)	163 (32.6%)	48 (22.4%)	57 (32.2%)	65 (27.2%)	
Job									<0.001
Agriculture	2,159 (39.5%)	645 (31.1%)	583 (45.2%)	503 (51.7%)	220 (44.0%)	46 (21.5%)	66 (37.3%)	96 (40.2%)	
Non-agriculture	1,495 (27.4%)	744 (35.9%)	401 (31.1%)	209 (21.5%)	64 (12.8%)	21 (9.8%)	20 (11.3%)	36 (15.1%)	
Retired	1,702 (31.1%)	649 (31.3%)	282 (21.8%)	246 (25.3%)	201 (40.2%)	136 (63.6%)	88 (49.7%)	100 (41.8%)	
Others	108 (2.0%)	33 (1.6%)	25 (1.9%)	14 (1.4%)	15 (3.0%)	11 (5.1%)	3 (1.7%)	7 (2.9%)	
Marital status									<0.001
In marriage	4,973 (91.0%)	1,945 (93.9%)	1,201 (93.0%)	877 (90.2%)	456 (91.2%)	155 (72.4%)	139 (78.5%)	200 (83.7%)	
Not in marriage	491 (9.0%)	126 (6.1%)	90 (7.0%)	95 (9.8%)	44 (8.8%)	59 (27.6%)	38 (21.5%)	39 (16.3%)	
Enabling factor									
Living arrangement									<0.001
Alone	246 (4.5%)	80 (3.9%)	41 (3.2%)	37 (3.8%)	19 (3.8%)	32 (15.0%)	17 (9.6%)	20 (8.4%)	
With others	5,218 (95.5%)	1,991 (96.1%)	1,250 (96.8%)	935 (96.2%)	481 (96.2%)	182 (85.0%)	160 (90.4%)	219 (91.6%)	
Type of residence									<0.001
Rural	3,188 (58.3%)	1,023 (49.4%)	785 (60.8%)	657 (67.6%)	343 (68.6%)	119 (55.6%)	111 (62.7%)	150 (62.8%)	
Urban	2,276 (41.7%)	1,048 (50.6%)	506 (39.2%)	315 (32.4%)	157 (31.4%)	95 (44.4%)	66 (37.3%)	89 (37.2%)	
Region									<0.001
Eastern	1,742 (31.9%)	707 (34.1%)	459 (35.6%)	278 (28.6%)	106 (21.2%)	68 (31.8%)	53 (29.9%)	71 (29.7%)	
Central	1,578 (28.9%)	650 (31.4%)	333 (25.8%)	260 (26.7%)	171 (34.2%)	45 (21.0%)	48 (27.1%)	71 (29.7%)	
Western	1,645 (30.1%)	543 (26.2%)	355 (27.5%)	350 (36.0%)	176 (35.2%)	83 (38.8%)	59 (33.3%)	79 (33.1%)	
Northeast	499 (9.1%)	171 (8.3%)	144 (11.2%)	84 (8.6%)	47 (9.4%)	18 (8.4%)	17 (9.6%)	18 (7.5%)	
Public health insurance									0.019
Yes	5,123 (93.8%)	1,948 (94.1%)	1,217 (94.3%)	910 (93.6%)	476 (95.2%)	196 (91.6%)	156 (88.1%)	220 (92.1%)	
No	341 (6.2%)	123 (5.9%)	74 (5.7%)	62 (6.4%)	24 (4.8%)	18 (8.4%)	21 (11.9%)	19 (7.9%)	
Household income per capita									<0.001
<1,000	3,074 (56.3%)	1,017 (49.1%)	692 (53.6%)	569 (58.5%)	344 (68.8%)	160 (74.8%)	118 (66.7%)	174 (72.8%)	
1,000+	2,390 (43.7%)	1,054 (50.9%)	599 (46.4%)	403 (41.5%)	156 (31.2%)	54 (25.2%)	59 (33.3%)	65 (27.2%)	
Health behavior									
Smoke behavior									<0.001
Yes	1,841 (33.7%)	734 (35.4%)	429 (33.2%)	284 (29.2%)	139 (27.8%)	80 (37.4%)	61 (34.5%)	114 (47.7%)	
No	3,623 (66.3%)	1,337 (64.6%)	862 (66.8%)	688 (70.8%)	361 (72.2%)	134 (62.6%)	116 (65.5%)	125 (52.3%)	
Drink behavior									<0.001
Yes	1,863 (34.1%)	812 (39.2%)	448 (34.7%)	262 (27.0%)	127 (25.4%)	65 (30.4%)	56 (31.6%)	93 (38.9%)	
No	3,601 (65.9%)	1,259 (60.8%)	843 (65.3%)	710 (73.0%)	373 (74.6%)	149 (69.6%)	121 (68.4%)	146 (61.1%)	

SH, sustainedly healthy; SP-ID: SP slightly improving followed by deterioration; CS-DG&L-SP, Cognitive status deteriorating gradually and lacking SP; ADLs-DI&L-SP, ADLs deteriorating followed by a slightly improvement and lacking SP; DIED-2, died in the second wave; DIED-3, died in the third wave; DIED-4, died in the fourth wave.

^a^*n* (%).

^b^Pearson’s Chi-squared test.

**Table 6:** Associations of baseline characteristics with the seven latent health transition classes by multivariable logistic regression.

Characteristic	SP-ID	CS-DG&L-SP	ADLs-DI&L-SP	DIED-2	DIED-3	DIED-4
	OR (95% CI)	OR (95% CI)	OR (95% CI)	OR (95% CI)	OR (95% CI)	OR (95% CI)
Predisposing factor						
Age						
45–54	—	—	—	—	—	—
55–64	1.12 (0.95, 1.33)	0.85 (0.70, 1.03)	1.86 (1.44, 2.40)^***^	1.81 (1.07, 3.04)^*^	1.29 (0.80, 2.07)	2.04 (1.33, 3.14)^**^
65+	1.03 (0.81, 1.31)	0.85 (0.65, 1.12)	2.26 (1.64, 3.12)^***^	6.64 (3.93, 11.2)^***^	4.53 (2.80, 7.34)^***^	5.01 (3.17, 7.91)^***^
Sex						
Male	—	—	—	—	—	—
Female	0.70 (0.58, 0.85)^***^	1.84 (1.46, 2.32)^***^	1.48 (1.13, 1.93)^**^	0.49 (0.33, 0.73)^***^	0.45 (0.29, 0.68)^***^	0.31 (0.20, 0.46)^***^
Education						
Illiterate	—	—	—	—	—	—
Literate or primary school	0.57 (0.39, 0.82)^**^	0.21 (0.15, 0.30)^***^	0.58 (0.38, 0.88)^*^	0.11 (0.07, 0.18)^***^	0.18 (0.11, 0.30)^***^	0.19 (0.12, 0.30)^***^
Junior high and above	0.39 (0.26, 0.56)^***^	0.06 (0.04, 0.08)^***^	0.28 (0.18, 0.44)^***^	0.04 (0.03, 0.08)^***^	0.10 (0.06, 0.17)^***^	0.08 (0.05, 0.13)^***^
Job						
Agriculture	—	—	—	—	—	—
Non-agriculture	0.73 (0.61, 0.88)^***^	0.67 (0.54, 0.84)^***^	0.54 (0.39, 0.74)^***^	0.98 (0.56, 1.73)	0.49 (0.29, 0.86)^*^	0.70 (0.46, 1.09)
Retired	0.53 (0.44, 0.65)^***^	0.64 (0.52, 0.80)^***^	1.13 (0.88, 1.46)	2.67 (1.79, 3.99)^***^	1.24 (0.84, 1.83)	1.13 (0.80, 1.59)
Others	0.91 (0.53, 1.57)	0.56 (0.29, 1.09)	1.34 (0.69, 2.60)	6.48 (2.84, 14.8)^***^	1.21 (0.35, 4.19)	2.34 (0.96, 5.72)
Marital status						
In marriage	—	—	—	—	—	—
Not in marriage	1.40 (1.00, 1.96)^*^	1.58 (1.10, 2.26)^*^	1.19 (0.76, 1.87)	2.82 (1.71, 4.66)^***^	2.85 (1.68, 4.84)^***^	2.23 (1.32, 3.76)^**^
Enabling factor						
Living arrangement						
Alone	—	—	—	—	—	—
With others	1.53 (0.96, 2.42)	1.49 (0.89, 2.49)	1.49 (0.79, 2.82)	1.04 (0.55, 1.96)	1.45 (0.70, 2.99)	1.35 (0.68, 2.69)
Type of residence						
Rural	—	—	—	—	—	—
Urban	0.79 (0.68, 0.93)^**^	0.63 (0.53, 0.76)^***^	0.46 (0.36, 0.58)^***^	0.79 (0.57, 1.10)	0.62 (0.43, 0.89)^**^	0.75 (0.55, 1.02)
Region						
Eastern	—	—	—	—	—	—
Central	0.77 (0.65, 0.93)^**^	1.06 (0.86, 1.32)	1.77 (1.35, 2.34)^***^	0.81 (0.53, 1.23)	1.01 (0.66, 1.53)	1.11 (0.78, 1.60)
Western	0.95 (0.79, 1.14)	1.57 (1.27, 1.94)^***^	1.96 (1.49, 2.59)^***^	1.57 (1.08, 2.28)^*^	1.33 (0.88, 1.99)	1.25 (0.87, 1.79)
Northeast	1.48 (1.14, 1.92)^**^	1.42 (1.03, 1.96)^*^	1.87 (1.25, 2.78)^**^	1.28 (0.71, 2.31)	1.55 (0.85, 2.82)	1.23 (0.70, 2.16)
Public health insurance						
Yes	—	—	—	—	—	—
No	1.01 (0.74, 1.37)	1.25 (0.89, 1.76)	0.88 (0.55, 1.40)	1.38 (0.78, 2.42)	2.22 (1.32, 3.74)^**^	1.49 (0.87, 2.54)
Household income per capita						
<1,000	—	—	—	—	—	—
1,000+	0.95 (0.81, 1.11)	0.97 (0.81, 1.16)	0.75 (0.59, 0.94)^*^	0.75 (0.52, 1.08)	0.96 (0.67, 1.38)	0.66 (0.47, 0.91)^*^
Health behavior						
Smoke behavior						
Yes	—	—	—	—	—	—
No	1.25 (1.05, 1.50)^*^	0.79 (0.63, 0.99)^*^	1.07 (0.82, 1.39)	0.89 (0.62, 1.28)	1.24 (0.85, 1.81)	0.83 (0.60, 1.14)
Drink behavior						
Yes	—	—	—	—	—	—
No	1.38 (1.16, 1.63)^***^	1.24 (1.01, 1.53)^*^	1.34 (1.04, 1.74)^*^	1.15 (0.80, 1.65)	1.28 (0.88, 1.86)	1.23 (0.90, 1.68)

*p < 0.05; **p < 0.01; ***p < 0.001.

SP-ID, SP slightly improving followed by deterioration; CS-DG&L-SP, Cognitive status deteriorating gradually and lacking SP; ADLs-DI&L-SP, ADLs deteriorating followed by a slightly improvement and lacking SP; DIED-2, died in the second wave; DIED-3, died in the third wave; DIED-4, died in the fourth wave; OR = odds ratio; CI = Confidence Interval.

The sustainedly healthy group is the reference group (*n* = 2071, 37.9%).

**Table 7:** Baseline characteristics of respondents in the seven classes after replacing health variables.

Characteristic	Overall, *N* = 5,455^a^	SH, *N* = 1,972^a^	SP-ID, *N* = 1,526^a^	CS-DG&L-SP, *N* = 944^a^	IADLs-DI&L-SP, *N* = 401^a^	DIED-2, *N* = 212^a^	DIED-3, *N* = 173^a^	DIED-4, *N* = 227^a^	*p*-value^b^
Predisposing factor									
Age									<0.001
45–54	2,221 (40.7%)	969 (49.1%)	677 (44.4%)	371 (39.3%)	120 (29.9%)	22 (10.4%)	31 (17.9%)	31 (13.7%)	
55–64	2,156 (39.5%)	724 (36.7%)	632 (41.4%)	411 (43.5%)	191 (47.6%)	59 (27.8%)	51 (29.5%)	88 (38.8%)	
65+	1,078 (19.8%)	279 (14.1%)	217 (14.2%)	162 (17.2%)	90 (22.4%)	131 (61.8%)	91 (52.6%)	108 (47.6%)	
Sex									<0.001
Male	3,000 (55.0%)	1,130 (57.3%)	918 (60.2%)	350 (37.1%)	178 (44.4%)	133 (62.7%)	114 (65.9%)	177 (78.0%)	
Female	2,455 (45.0%)	842 (42.7%)	608 (39.8%)	594 (62.9%)	223 (55.6%)	79 (37.3%)	59 (34.1%)	50 (22.0%)	
Education									<0.001
Illiterate	533 (9.8%)	54 (2.7%)	75 (4.9%)	226 (23.9%)	38 (9.5%)	63 (29.7%)	35 (20.2%)	42 (18.5%)	
Literate or primary school	2,431 (44.6%)	698 (35.4%)	689 (45.2%)	515 (54.6%)	222 (55.4%)	101 (47.6%)	83 (48.0%)	123 (54.2%)	
Junior high and above	2,491 (45.7%)	1,220 (61.9%)	762 (49.9%)	203 (21.5%)	141 (35.2%)	48 (22.6%)	55 (31.8%)	62 (27.3%)	
Job									<0.001
Agriculture	2,153 (39.5%)	621 (31.5%)	662 (43.4%)	512 (54.2%)	155 (38.7%)	46 (21.7%)	64 (37.0%)	93 (41.0%)	
Non-agriculture	1,504 (27.6%)	701 (35.5%)	489 (32.0%)	177 (18.8%)	61 (15.2%)	21 (9.9%)	20 (11.6%)	35 (15.4%)	
Retired	1,692 (31.0%)	618 (31.3%)	348 (22.8%)	238 (25.2%)	176 (43.9%)	134 (63.2%)	86 (49.7%)	92 (40.5%)	
Others	106 (1.9%)	32 (1.6%)	27 (1.8%)	17 (1.8%)	9 (2.2%)	11 (5.2%)	3 (1.7%)	7 (3.1%)	
Marital status									<0.001
In marriage	4,969 (91.1%)	1,845 (93.6%)	1,424 (93.3%)	860 (91.1%)	360 (89.8%)	155 (73.1%)	136 (78.6%)	189 (83.3%)	
Not in marriage	486 (8.9%)	127 (6.4%)	102 (6.7%)	84 (8.9%)	41 (10.2%)	57 (26.9%)	37 (21.4%)	38 (16.7%)	
Enabling factor									
Living arrangement									<0.001
Alone	245 (4.5%)	82 (4.2%)	49 (3.2%)	28 (3.0%)	18 (4.5%)	31 (14.6%)	17 (9.8%)	20 (8.8%)	
With others	5,210 (95.5%)	1,890 (95.8%)	1,477 (96.8%)	916 (97.0%)	383 (95.5%)	181 (85.4%)	156 (90.2%)	207 (91.2%)	
Type of residence									<0.001
Rural	3,183 (58.4%)	982 (49.8%)	927 (60.7%)	655 (69.4%)	250 (62.3%)	118 (55.7%)	108 (62.4%)	143 (63.0%)	
Urban	2,272 (41.6%)	990 (50.2%)	599 (39.3%)	289 (30.6%)	151 (37.7%)	94 (44.3%)	65 (37.6%)	84 (37.0%)	
Region									<0.001
Eastern	1,736 (31.8%)	662 (33.6%)	538 (35.3%)	253 (26.8%)	98 (24.4%)	66 (31.1%)	52 (30.1%)	67 (29.5%)	
Central	1,562 (28.6%)	623 (31.6%)	405 (26.5%)	257 (27.2%)	120 (29.9%)	45 (21.2%)	46 (26.6%)	66 (29.1%)	
Western	1,659 (30.4%)	533 (27.0%)	423 (27.7%)	348 (36.9%)	137 (34.2%)	83 (39.2%)	58 (33.5%)	77 (33.9%)	
Northeast	498 (9.1%)	154 (7.8%)	160 (10.5%)	86 (9.1%)	46 (11.5%)	18 (8.5%)	17 (9.8%)	17 (7.5%)	
Public health insurance									0.019
Yes	5,115 (93.8%)	1,853 (94.0%)	1,439 (94.3%)	885 (93.8%)	382 (95.3%)	194 (91.5%)	152 (87.9%)	210 (92.5%)	
No	340 (6.2%)	119 (6.0%)	87 (5.7%)	59 (6.2%)	19 (4.7%)	18 (8.5%)	21 (12.1%)	17 (7.5%)	
Household income per capita									<0.001
<1,000	3,057 (56.0%)	977 (49.5%)	797 (52.2%)	573 (60.7%)	272 (67.8%)	158 (74.5%)	116 (67.1%)	164 (72.2%)	
1,000+	2,398 (44.0%)	995 (50.5%)	729 (47.8%)	371 (39.3%)	129 (32.2%)	54 (25.5%)	57 (32.9%)	63 (27.8%)	
Health behavior									
Smoke behavior									<0.001
Yes	1,824 (33.4%)	684 (34.7%)	513 (33.6%)	274 (29.0%)	108 (26.9%)	81 (38.2%)	59 (34.1%)	105 (46.3%)	
No	3,631 (66.6%)	1,288 (65.3%)	1,013 (66.4%)	670 (71.0%)	293 (73.1%)	131 (61.8%)	114 (65.9%)	122 (53.7%)	
Drink behavior									<0.001
Yes	1,852 (34.0%)	768 (38.9%)	535 (35.1%)	242 (25.6%)	96 (23.9%)	66 (31.1%)	56 (32.4%)	89 (39.2%)	
No	3,603 (66.0%)	1,204 (61.1%)	991 (64.9%)	702 (74.4%)	305 (76.1%)	146 (68.9%)	117 (67.6%)	138 (60.8%)	

SH, sustainedly healthy; SP-ID, SP slightly improving followed by deterioration; CS-DG&L-SP, Cognitive status deteriorating gradually and lacking SP; IADLs-DI&L-SP, IADLs deteriorating followed by a slightly improvement and lacking SP; DIED-2, died in the second wave; DIED-3, died in the third wave; DIED-4, died in the fourth wave.

^a^*n* (%).

^b^Pearson’s Chi-squared test.

**Table 8:** Associations of baseline characteristics with the seven classes after replacing health variables.

Characteristic	SP-ID	CS-DG&L-SP	IADLs-DI&L-SP	DIED-2	DIED-3	DIED-4
	OR (95% CI)	OR (95% CI)	OR (95% CI)	OR (95% CI)	OR (95% CI)	OR (95% CI)
Predisposing factor						
Age						
45–54	—	—	—	—	—	—
55–64	1.10 (0.94, 1.29)	1.01 (0.83, 1.24)	1.52 (1.16, 1.99)^**^	1.89 (1.12, 3.19)^*^	1.36 (0.84, 2.19)	2.15 (1.38, 3.34)^***^
65+	1.03 (0.82, 1.30)	1.12 (0.85, 1.47)	1.62 (1.14, 2.30)^**^	6.89 (4.08, 11.6)^***^	4.76 (2.92, 7.77)^***^	5.39 (3.37, 8.63)^***^
Sex						
Male	—	—	—	—	—	—
Female	0.71 (0.59, 0.85)^***^	1.93 (1.52, 2.45)^***^	1.24 (0.92, 1.66)	0.48 (0.32, 0.72)^***^	0.45 (0.30, 0.69)^***^	0.29 (0.20, 0.44)^***^
Education						
Illiterate	—	—	—	—	—	—
Literate or primary school	0.68 (0.47, 0.99)^*^	0.21 (0.15, 0.29)^***^	0.52 (0.33, 0.81)^**^	0.12 (0.07, 0.18)^***^	0.18 (0.11, 0.30)^***^	0.18 (0.11, 0.29)^***^
Junior high and above	0.48 (0.33, 0.70)^***^	0.06 (0.04, 0.08)^***^	0.26 (0.16, 0.41)^***^	0.05 (0.03, 0.08)^***^	0.10 (0.06, 0.17)^***^	0.08 (0.05, 0.13)^***^
Job						
Agriculture	—	—	—	—	—	—
Non-agriculture	0.78 (0.65, 0.93)^**^	0.61 (0.48, 0.76)^***^	0.65 (0.46, 0.92)^*^	0.98 (0.55, 1.73)	0.51 (0.30, 0.89)^*^	0.71 (0.46, 1.11)
Retired	0.60 (0.49, 0.72)^***^	0.61 (0.49, 0.75)^***^	1.31 (1.00, 1.72)	2.65 (1.78, 3.97)^***^	1.24 (0.84, 1.84)	1.05 (0.74, 1.50)
Others	0.87 (0.51, 1.48)	0.63 (0.34, 1.20)	1.07 (0.49, 2.34)	6.23 (2.73, 14.2)^***^	1.17 (0.33, 4.08)	2.32 (0.94, 5.69)
Marital status						
In marriage	—	—	—	—	—	—
Not in marriage	1.24 (0.90, 1.72)	1.37 (0.95, 1.98)	1.45 (0.92, 2.30)	2.64 (1.59, 4.39)^***^	2.67 (1.56, 4.57)^***^	2.16 (1.27, 3.67)^**^
Enabling factor						
Living arrangement						
Alone	—	—	—	—	—	—
With others	1.49 (0.97, 2.30)	2.05 (1.19, 3.56)^*^	1.67 (0.87, 3.18)	1.13 (0.59, 2.14)	1.51 (0.73, 3.12)	1.36 (0.68, 2.72)
Type of residence						
Rural	—	—	—	—	—	—
Urban	0.77 (0.67, 0.90)^***^	0.61 (0.50, 0.74)^***^	0.61 (0.48, 0.78)^***^	0.82 (0.58, 1.14)	0.64 (0.44, 0.92)^*^	0.77 (0.56, 1.06)
Region						
Eastern	—	—	—	—	—	—
Central	0.78 (0.66, 0.93)^**^	1.15 (0.92, 1.43)	1.30 (0.97, 1.75)	0.80 (0.52, 1.22)	0.95 (0.62, 1.45)	1.05 (0.72, 1.53)
Western	0.93 (0.78, 1.11)	1.63 (1.32, 2.03)^***^	1.57 (1.17, 2.11)^**^	1.53 (1.05, 2.23)^*^	1.26 (0.84, 1.91)	1.23 (0.86, 1.78)
Northeast	1.46 (1.13, 1.89)^**^	1.73 (1.25, 2.41)^**^	2.12 (1.41, 3.19)^***^	1.41 (0.78, 2.56)	1.68 (0.92, 3.06)	1.34 (0.75, 2.40)
Public health insurance						
Yes	—	—	—	—	—	—
No	1.00 (0.74, 1.34)	1.24 (0.87, 1.76)	0.79 (0.47, 1.31)	1.39 (0.79, 2.45)	2.27 (1.34, 3.84)^**^	1.39 (0.80, 2.43)
Household income per capita						
<1,000	—	—	—	—	—	—
1,000+	1.00 (0.86, 1.16)	0.95 (0.79, 1.14)	0.73 (0.57, 0.93)^*^	0.76 (0.53, 1.10)	0.96 (0.67, 1.38)	0.69 (0.49, 0.97)^*^
Health behavior						
Smoke behavior						
Yes	—	—	—	—	—	—
No	1.19 (1.00, 1.41)^*^	0.73 (0.58, 0.92)^**^	1.07 (0.80, 1.43)	0.86 (0.60, 1.23)	1.23 (0.84, 1.81)	0.88 (0.63, 1.21)
Drink behavior						
Yes	—	—	—	—	—	—
No	1.37 (1.16, 1.61)^***^	1.33 (1.08, 1.65)^**^	1.55 (1.16, 2.06)^**^	1.15 (0.80, 1.66)	1.24 (0.85, 1.81)	1.23 (0.89, 1.70)

*p < 0.05; **p < 0.01; ***p < 0.001.

SP-ID, SP slightly improving followed by deterioration; CS-DG&L-SP, Cognitive status deteriorating gradually and lacking SP; IADLs-DI&L-SP, IADLs deteriorating followed by a slightly improvement and lacking SP; DIED-2, died in the second wave; DIED-3, died in the third wave; DIED-4, died in the fourth wave; OR = odds ratio; CI = Confidence Interval.

The sustainedly healthy group is the reference group (*n* = 1972, 36.2%).

**Table 9:** Baseline characteristics of respondents in the five groups after replacing health variables.

Characteristic	Overall, *N* = 5,455^a^	SH, *N* = 1,972^a^	SP-ID, *N* = 1,526^a^	CS-DG&L-SP, *N* = 944^a^	IADLs-DI&L-SP, *N* = 401^a^	DIFP, *N* = 612^a^	*p*-value^b^
Predisposing factor							
Age							<0.001
45–54	2,221 (40.7%)	969 (49.1%)	677 (44.4%)	371 (39.3%)	120 (29.9%)	84 (13.7%)	
55–64	2,156 (39.5%)	724 (36.7%)	632 (41.4%)	411 (43.5%)	191 (47.6%)	198 (32.4%)	
65+	1,078 (19.8%)	279 (14.1%)	217 (14.2%)	162 (17.2%)	90 (22.4%)	330 (53.9%)	
Sex							<0.001
Male	3,000 (55.0%)	1,130 (57.3%)	918 (60.2%)	350 (37.1%)	178 (44.4%)	424 (69.3%)	
Female	2,455 (45.0%)	842 (42.7%)	608 (39.8%)	594 (62.9%)	223 (55.6%)	188 (30.7%)	
Education							<0.001
Illiterate	533 (9.8%)	54 (2.7%)	75 (4.9%)	226 (23.9%)	38 (9.5%)	140 (22.9%)	
Literate or primary school	2,431 (44.6%)	698 (35.4%)	689 (45.2%)	515 (54.6%)	222 (55.4%)	307 (50.2%)	
Junior high and above	2,491 (45.7%)	1,220 (61.9%)	762 (49.9%)	203 (21.5%)	141 (35.2%)	165 (27.0%)	
Job							<0.001
Agriculture	2,153 (39.5%)	621 (31.5%)	662 (43.4%)	512 (54.2%)	155 (38.7%)	203 (33.2%)	
Non-agriculture	1,504 (27.6%)	701 (35.5%)	489 (32.0%)	177 (18.8%)	61 (15.2%)	76 (12.4%)	
Retired	1,692 (31.0%)	618 (31.3%)	348 (22.8%)	238 (25.2%)	176 (43.9%)	312 (51.0%)	
Others	106 (1.9%)	32 (1.6%)	27 (1.8%)	17 (1.8%)	9 (2.2%)	21 (3.4%)	
Marital status							<0.001
In marriage	4,969 (91.1%)	1,845 (93.6%)	1,424 (93.3%)	860 (91.1%)	360 (89.8%)	480 (78.4%)	
Not in marriage	486 (8.9%)	127 (6.4%)	102 (6.7%)	84 (8.9%)	41 (10.2%)	132 (21.6%)	
Enabling factor							
Living arrangement							<0.001
Alone	245 (4.5%)	82 (4.2%)	49 (3.2%)	28 (3.0%)	18 (4.5%)	68 (11.1%)	
With others	5,210 (95.5%)	1,890 (95.8%)	1,477 (96.8%)	916 (97.0%)	383 (95.5%)	544 (88.9%)	
Type of residence							<0.001
Rural	3,183 (58.4%)	982 (49.8%)	927 (60.7%)	655 (69.4%)	250 (62.3%)	369 (60.3%)	
Urban	2,272 (41.6%)	990 (50.2%)	599 (39.3%)	289 (30.6%)	151 (37.7%)	243 (39.7%)	
Region							<0.001
Eastern	1,736 (31.8%)	662 (33.6%)	538 (35.3%)	253 (26.8%)	98 (24.4%)	185 (30.2%)	
Central	1,562 (28.6%)	623 (31.6%)	405 (26.5%)	257 (27.2%)	120 (29.9%)	157 (25.7%)	
Western	1,659 (30.4%)	533 (27.0%)	423 (27.7%)	348 (36.9%)	137 (34.2%)	218 (35.6%)	
Northeast	498 (9.1%)	154 (7.8%)	160 (10.5%)	86 (9.1%)	46 (11.5%)	52 (8.5%)	
Public health insurance							0.023
Yes	5,115 (93.8%)	1,853 (94.0%)	1,439 (94.3%)	885 (93.8%)	382 (95.3%)	556 (90.8%)	
No	340 (6.2%)	119 (6.0%)	87 (5.7%)	59 (6.2%)	19 (4.7%)	56 (9.2%)	
Household income per capita							<0.001
<1,000	3,057 (56.0%)	977 (49.5%)	797 (52.2%)	573 (60.7%)	272 (67.8%)	438 (71.6%)	
1,000+	2,398 (44.0%)	995 (50.5%)	729 (47.8%)	371 (39.3%)	129 (32.2%)	174 (28.4%)	
Health behavior							
Smoke behavior							<0.001
Yes	1,824 (33.4%)	684 (34.7%)	513 (33.6%)	274 (29.0%)	108 (26.9%)	245 (40.0%)	
No	3,631 (66.6%)	1,288 (65.3%)	1,013 (66.4%)	670 (71.0%)	293 (73.1%)	367 (60.0%)	
Drink behavior							<0.001
Yes	1,852 (34.0%)	768 (38.9%)	535 (35.1%)	242 (25.6%)	96 (23.9%)	211 (34.5%)	
No	3,603 (66.0%)	1,204 (61.1%)	991 (64.9%)	702 (74.4%)	305 (76.1%)	401 (65.5%)	

SH, sustainedly healthy; SP-ID, SP slightly improving followed by deterioration; CS-DG&L-SP, Cognitive status deteriorating gradually and lacking SP; IADLs-DI&L-SP, IADLs deteriorating followed by a slightly improvement and lacking SP; DIFP, died in the follow-up period.

^a^*n* (%).

^b^Pearson’s Chi-squared test.

**Table 10:** Associations of baseline characteristics with the five groups after replacing health variables.

Characteristic	SP-ID	CS-DG&L-SP	IADLs-DI&L-SP	DIFP
	OR (95% CI)	OR (95% CI)	OR (95% CI)	OR (95% CI)
Predisposing factor				
Age				
45–54	—	—	—	—
55–64	1.10 (0.94, 1.29)	1.01 (0.83, 1.24)	1.52 (1.16, 1.99)^**^	1.79 (1.34, 2.40)^***^
65+	1.03 (0.82, 1.30)	1.11 (0.85, 1.46)	1.62 (1.14, 2.29)^**^	5.60 (4.09, 7.66)^***^
Sex				
Male	—	—	—	—
Female	0.71 (0.59, 0.85)^***^	1.93 (1.52, 2.45)^***^	1.23 (0.92, 1.65)	0.40 (0.30, 0.53)^***^
Education				
Illiterate	—	—	—	—
Literate or primary school	0.68 (0.47, 0.98)^*^	0.21 (0.15, 0.29)^***^	0.52 (0.33, 0.81)^**^	0.15 (0.11, 0.22)^***^
Junior high and above	0.48 (0.33, 0.70)^***^	0.06 (0.04, 0.08)^***^	0.26 (0.16, 0.41)^***^	0.07 (0.05, 0.10)^***^
Job				
Agriculture	—	—	—	—
Non-agriculture	0.78 (0.65, 0.93)^**^	0.61 (0.48, 0.76)^***^	0.65 (0.46, 0.92)^*^	0.71 (0.51, 0.97)^*^
Retired	0.60 (0.49, 0.72)^***^	0.60 (0.48, 0.75)^***^	1.31 (0.99, 1.72)	1.47 (1.14, 1.89)^**^
Others	0.86 (0.51, 1.47)	0.62 (0.33, 1.18)	1.06 (0.49, 2.32)	2.77 (1.46, 5.24)^**^
Marital status				
In marriage	—	—	—	—
Not in marriage	1.24 (0.90, 1.72)	1.36 (0.94, 1.97)	1.45 (0.92, 2.29)	2.49 (1.72, 3.62)^***^
Enabling factor				
Living arrangement				
Alone	—	—	—	—
With others	1.49 (0.96, 2.29)	2.05 (1.19, 3.56)^*^	1.67 (0.88, 3.19)	1.31 (0.81, 2.14)
Type of residence				
Rural	—	—	—	—
Urban	0.77 (0.67, 0.90)^***^	0.61 (0.50, 0.74)^***^	0.61 (0.48, 0.78)^***^	0.74 (0.59, 0.92)^**^
Region				
Eastern	—	—	—	—
Central	0.78 (0.66, 0.93)^**^	1.15 (0.92, 1.43)	1.31 (0.97, 1.75)	0.94 (0.72, 1.22)
Western	0.93 (0.78, 1.11)	1.63 (1.31, 2.03)^***^	1.57 (1.17, 2.11)^**^	1.33 (1.03, 1.72)^*^
Northeast	1.46 (1.13, 1.89)^**^	1.73 (1.25, 2.40)^**^	2.12 (1.41, 3.18)^***^	1.46 (0.98, 2.15)
Public health insurance				
Yes	—	—	—	—
No	1.00 (0.74, 1.34)	1.24 (0.87, 1.76)	0.79 (0.47, 1.31)	1.64 (1.12, 2.38)^*^
Household income per capita				
<1,000	—	—	—	—
1,000+	1.00 (0.86, 1.16)	0.95 (0.79, 1.14)	0.73 (0.57, 0.93)^*^	0.79 (0.63, 0.99)^*^
Health behavior				
Smoke behavior				
Yes	—	—	—	—
No	1.19 (1.00, 1.41)^*^	0.73 (0.58, 0.92)^**^	1.07 (0.80, 1.44)	0.96 (0.75, 1.21)
Drink behavior				
Yes	—	—	—	—
No	1.37 (1.16, 1.61)^***^	1.33 (1.08, 1.65)^**^	1.55 (1.16, 2.06)^**^	1.21 (0.96, 1.53)

SP-ID, SP slightly improving followed by deterioration; CS-DG&L-SP, Cognitive status deteriorating gradually and lacking SP; IADLs-DI&L-SP, IADLs deteriorating followed by a slightly improvement and lacking SP; DIFP, died in the follow-up period; OR = odds ratio; CI = Confidence Interval.

The sustainedly healthy group is the reference group (*n* = 1972, 36.2%).

^*^p < 0.05; ^**^p < 0.01; ^***^p < 0.001.

## 4 Discussion

We identified five distinct health transition pattern groups during 2011–2018 for the middle-aged and elderly population in China using the person-centered approach of RMLCA. The RMLCA approach is similar to the growth mixture modeling, but the former does not consider the particular functional relationship between each latent class and time and can observe latent transitions across multiple dimensions separately (17). Based on this, we systematically integrated multidimensional health indicators to provide greater insight into individual health transition patterns, advancing previous trajectory studies about single health dimensions (7, 12).

In particular, except for physical and psychological dimensions (14, 15), a social dimension was introduced in this study, which characterized one of the identified health transition groups (the SP-ID group). Similar to static studies based on cross-sectional data (11), our study showed noticeable differences in the physical, psychological, and social dimensions. Most participants assigned to the SP-ID group lacked SP during the follow-up period and tended to improve and then deteriorate. However, their physical and psychological health status remained good and stable. The ADLs-DI&L-SP and CS-DG&L-SP groups exhibited similar proportions and trends in the social dimensions of health. The literature similarly demonstrated a complex reciprocal relationship between SP and cognitive problems or physical functions (40, 41). It indicated that introducing the social dimension was valuable for assessing the heterogeneous health transition patterns in the middle-aged and older Chinese population. Moreover, these findings showed that deterioration in the social dimension of health did not necessarily deteriorate physical and psychological health; however, poor physical or psychological health might lead to a decline in SP. The positive effect of SP on maintaining physical and psychological health has been largely confirmed in previous studies (42, 43). Therefore, it is important to pay attention to not only the physical or psychological health but also the social dimensions of the health of older people who are in adverse physical or psychological health conditions.

Furthermore, in contrast to previous studies (7, 15), we also observed fluctuating health states (the ADLs-DI&L-SP and SP-ID groups), i.e., the possibility of a transient recovery of health status over time, which provides evidence for healthy aging policy and practice. One of the patterns that fluctuated was the SP-ID group, where individuals showed an improvement in SP followed by deterioration, indicating a possibility of improvement in the social dimension of health, similar to a previous trajectory study of social engagement (12). Another group with significant upward and downward fluctuations in the pattern of health transition was the ADLs-DI&L-SP group. Previous studies have found that positive beliefs about older people as a non-frail category would be helpful for recovery from ADLs disabilities (44, 45). These findings provide evidence for healthy aging policy and practice.

The disparities in health transition patterns could be attributed to predisposing factors, enabling factors, and health behaviors in the Andersen’s model, especially for sex, education, job, type of residence, and region.

Compared to females, the males were more likely to be assigned to the SP-ID and DIFP groups, while the females were more likely to deteriorate in physical and psychological health. This reaffirms the existence of the “gender paradox” again; i.e., women have lower health and death rates than men (46). In terms of SP, females may have the advantage of providing more care for others (47), given that it is more common for females to take care of their grandchildren in China (48). Zhang et al. have pointed out that males enjoy more health benefits from providing care to their grandchildren than females (49). Hence, more attention should be paid to the improvement of SP of elderly men, such as advocating that they provide more care of grandchildren, in order to promote elderly men health not only in social dimension but also in all dimensions. According to previous studies, females are more likely to experience cognitive impairment (50, 51) because they retire at a younger age than males in China (49). Similarly, many studies have shown females in poor physical condition (52, 53), possibly because their biological advantages earlier in life are lost in old age (51). Therefore, more interventions are necessary for older males to reduce social isolation and focus on life-threatening health problems, and addressing physical and psychological health problems in females should start at an early stage of life.

Individuals with higher educational levels were less likely to experience deterioration in all dimensions of health, with the most significant impact on cognitive ability (15, 54). In addition, the effect of education on the overall health status of older people may be influenced by other potential mechanisms related to psychological health, such as stress level (55). In older people, it is more difficult to re-advance their education. However, studies have shown that lifelong learning can reduce the impact of a lack of formal education on health in the middle to later adulthood, with a particular impact on psychological health (56). Therefore, university-based lifelong learning programs offer a viable option for enhancing educational attainment (57). Community-based education for older people, particularly health education, can increase the social trust of older people and achieve the goal of an age-friendly society (58). The internet can make learning opportunities more accessible to older people (59). Therefore, China has introduced a series of measures to broaden middle-aged and older senior citizens’ access to education, such as the establishment of the Seniors University of China (60) and the special action plan for ageing and barrier-free transformation of Internet applications (61).

Concerning working status, individuals working in the agriculture sector experienced more deterioration in all the dimensions of health status and even death than those in the non-agriculture sector, as evidenced by studies from China and Italy (62, 63). Therefore, it alerts policymakers for the health status of people in different occupations, especially those in the agriculture sector. Consistent with previous research findings, individuals who were retired at the base time or unemployed and those who never worked were more likely to die during the follow-up period (64). China’s policy of raising the retirement age in progressive steps has supported the employment of middle-aged and older people. However, there is also a risk that more people will become unemployed. Therefore, an age-friendly employment scheme is required to prevent age discrimination among middle-aged and older people. It is also important to fully respect the wishes of older people to re-join the workforce and promote their health in multiple ways.

Middle-aged and older people living in rural areas had a higher risk of multidimensional health deterioration and even death, consistent with previous studies (65–67). Despite the completion of merging the rural new cooperative medical scheme and urban resident-based basic medical insurance scheme and the adherence to the strategic approach of broad health insurance coverage in China, there is still a significant gap in the accessibility of health services between urban and rural areas (68). Expanding investment in health care in rural areas and strengthening the rural health workforce by the government have effectively reduced the rural–urban health gap (69).

People living in the eastern regions generally had better health on all dimensions than those in other regions, which was particularly evident in their physical health. This finding is inextricably linked to the relatively good economic conditions and the relative surplus of medical resources in the eastern part of China (70, 71). In fact, there are differences in specific medical security policies between different regions, with faster-developing regions likely to have higher reimbursement rates from health insurance systems (72). The subsequent reform should allocate medical and health resources and optimize medical security policies more precisely according to different regions’ population distribution and economic development. In addition, attempts can be made to promote the intra-regional sharing of medical and internet resources to achieve support between different regions. Notably, residents in the central region had a higher level of SP than those in the eastern region, which might be related to the much faster pace of development in eastern China, some extent squeezing the SP of middle-aged and older people. For the SP-ID group living in eastern China, it calls for multi-channel social participation network and the promotion of community-based engagement programs as a targeted intervention.

There are still some limitations. Firstly, although the complete case analysis method including only samples with complete data for analysis is common used in previous studies of the CHARLS database (36, 37), there were the potential selection bias in this study. Secondly, to present the model with relative clarity, we did not include more measurable health indicators, such as combination of ADL and IADL, chronic diseases, depressive factors (like the Center for Epidemiologic Studies Depression scale), and self-rated health. Thirdly, factors such as propensity, enablement, and health behaviors were measured using baseline data rather than as time-varying variable, to reduce sample bias caused by more missing data and the complication of analyses. Finally, for the stratification for household income per capita, we chose the figure (1,000 yuan) closest to the median of the sample in the thousands of yuan, and the criteria were not objective enough. A representative sample with more complete information and the changes of determinants should be considered in further research.

## 5 Conclusion

In conclusion, combining social dimension with physical and psychological dimensions, we identified five health transition patterns among Chinese middle-aged and older adults. This study suggests that it is crucial to identify different types of health dynamics, particularly focusing on the social dimensions of health status and the presence of fluctuating states. Andersen’s health service model showed different influencing factors for different latent health transition patterns. This study provides new insights into the complex dynamic processes and heterogeneous health of middle-aged and older people in China. The findings are valuable for personalized health services and healthy aging strategies.

## Data Availability

Publicly available datasets were analyzed in this study. This data can be found at: https://charls.pku.edu.cn/.
